# Vancomycin-induced thrombocytopenia in a 60-year-old man: a case report

**DOI:** 10.4076/1752-1947-3-7290

**Published:** 2009-06-26

**Authors:** Ravish A Shah, Adnan Musthaq, Nancy Khardori

**Affiliations:** 1 Division of Infectious Diseases, Department of Internal Medicine, Southern Illinois University School of Medicine, Springfield, IL, USA

## Abstract

**Introduction:**

Vancomycin, a glycopeptide antibiotic, is used to treat resistant gram-positive infections. There has been a 10- to 20-fold increase in its use over the past 25 years. Although ototoxicity and nephrotoxicity are well known side effects of vancomycin, it can also induce platelet reactive antibodies leading to life-threatening thrombocytopenia. Vancomycin is often clinically overlooked as a cause of thrombocytopenia, especially in a scenario of sepsis or when there is use of heparin. We report a proven case of vancomycin-induced thrombocytopenia and its reversal after discontinuation of vancomycin.

**Case presentation:**

A 60-year-old man with a history of hypertension, congestive heart failure and dyslipidemia was admitted for a right shoulder rotator cuff tear. He underwent right-shoulder arthroscopy and rotator cuff repair. About three weeks later, he developed pain, swelling and purulent drainage from his right shoulder. Arthroscopic irrigation and drainage was then performed. Intraoperative fluid revealed the presence of *Methicillin susceptible Staphylococcus aureus*, vancomycin-sensitive *Enterococcus spp*. and *Serratia marcescens*. The patient had no known allergies. After reviewing his antimicrobial susceptibility, he was started on vancomycin 1500 mgs intravenously every 12 hours (to treat both *Staphylococcus aureus* and *Enterococcus spp*) and ciprofloxacin 750 mgs by oral induction every 12 hours. The patient's condition improved following antibiotic treatment. He was discharged and allowed to go home on IV vancomycin and oral ciprofloxacin. The patient's platelet count on the day of starting vancomycin therapy was 253 × 10^3^/mm^3^. At weeks one, two and three, the counts were 231 × 10^3^/mm^3^, 272 × 10^3^/mm and 6 × 103/mm^3^, respectively. The patient was admitted for further work-up of the thrombocytopenia. He was later shown to have vancomycin-induced platelet-reactive antibodies, causing significant thrombocytopenia, and then reversal after his vancomycin medication was discontinued.

**Conclusion:**

Thrombocytopenia is a potentially life-threatening condition. Vancomycin is often clinically overlooked as a cause of thrombocytopenia, especially in a scenario of sepsis or when there is use of heparin. Simple laboratory testing with drug-dependent antibodies can be helpful in identifying vancomycin as a cause of thrombocytopenia.

## Introduction

Vancomycin, a glycopeptide antibiotic, is used to treat resistant gram-positive infections [1]-[5]. Recent studies show a 10- to 20-fold increase in its use over the past 25 years [5]-[7]. Nephrotoxicity (associated with elevated trough levels), ototoxicity associated with elevated peak levels, red-man syndrome and reversible neutropenia are well known side effects of vancomycin. Thrombocytopenia related to vancomycin is rarely reported in the literature [1,8]-[11]. This may be due to co-morbid conditions and/or concurrent medications. Furthermore, vancomycin as a cause of thrombocytopenia may be overlooked, especially in a scenario of sepsis or when heparin is used [5,12]-[14]. Drygalski et al. [5] reported that vancomycin can induce platelet-reactive antibodies, leading to thrombocytopenia and life-threatening bleeding complications. We report a proven case of vancomycin-induced platelet-reactive antibodies causing significant thrombocytopenia, and then reversal after discontinuation of vancomycin medication.

## Case presentation

A 60-year-old man with a history of hypertension, congestive heart failure, dyslipidemia and gastro-esophageal reflux disease was admitted for right shoulder rotator cuff tear. His medications included spironolactone 25 mg once daily, aspirin 325 mg once daily, pantoprazole 40 mg once daily, olmesartan 40 mg once daily, colesevelam 625 mg three times a day, furosemide 20 mg once daily and ezetimibe 10 mg once daily. The patient then underwent right shoulder arthroscopy and rotator cuff repair.

The postoperative course was uneventful until three weeks later, when he developed pain, swelling and a purulent drainage from his right shoulder. The patient returned to the emergency department and an arthroscopic irrigation and drainage was performed. Intraoperative fluid was sent for gram stain, culture and sensitivity, which later revealed the presence of* Methicillin susceptible staphylococcus aureus*, vancomycin-sensitive *Enterococcus spp*. and *Serratia marcescens*.

The patient had no known allergies. After reviewing antimicrobial susceptibility, he was started on vancomycin 1500 mgs intravenously (IV) every 12 hours (to treat both *Staphylococcus aureus* and *Enterococcus spp*) and ciprofloxacin 750 mgs by oral induction every 12 hours. The patient's vancomycin peak level was at 36.2 mcg/ml, and the trough level was at 14.7 mcg/ml after the third dose. The patient's shoulder pain and swelling improved after antibiotic treatment. He was discharged and allowed to go home on IV vancomycin and oral ciprofloxacin. His platelet count on the day he was started on vancomycin therapy was 253 × 10^3^/mm^3^ (the normal range is 150 to 400 × 10^3^/mm^3^). His complete blood count, vancomycin peak and trough levels were monitored in the infectious disease clinic. The platelet count was 231 × 10^3^/mm^3^ in the first week and 272 × 10^3^/mm^3^ in the second week. The platelet count was 6 × 10^3^/mm^3^ in the third week after treatment.

The patient was admitted for further work-up of thrombocytopenia. In addition to vancomycin and ciprofloxacin, he received heparin flushes (50 units daily) for three weeks to maintain potency of the peripherally inserted central catheter (PICC). In the emergency department, his vital signs were reported as 37.4 degrees Celsius temperature, pulse of 88 per minute, respiratory rate of 18 per minute and blood pressure of 130/80 mmHg. Further examination revealed the presence of extensive petechial hemorrhages on both of his lower extremities. Peripheral pulses were present and equal in all extremities. A repeat platelet count in the emergency department yielded a result of 9 × 10^3^/mm^3^.

On the first day after admission, administration of aspirin, vancomycin and heparin flushes of the PICC on the patient were discontinued. As part of his thrombocytopenia work-up, blood cultures, a disseminated intravascular coagulation panel and heparin-induced antiplatelet antibodies were obtained. Given the patient's presentation, vancomycin-induced anti-platelet antibodies were also ordered - this was done at the Blood Center of the Wisconsin Platelet and Neutrophil Immunology Laboratory, using immunofluorescence flow cytometry. These results were reviewed three days after the blood specimen was drawn. During this time, the patient received one unit of platelet transfusion, which raised his platelet count from 9 × 10^3^/mm^3^ to 28 × 10^3^/mm^3^. On the second day, the patient was given another unit of platelet transfusion, which further elevated his platelet count to 60 × 10^3^/mm^3^. By the third day, his platelet count had increased to 271 × 10^3^/mm^3^. He was discharged home on an initial dose of 100 mg intravenous tigecycline, followed by 50 mg IV every 12 hours. The patient continued with a follow-up at the infectious disease clinic and his platelet count was already at 344 × 10^3^/mm^3^ after one week and 338 × 10^3^ /mm^3^ after two weeks.

The differential diagnoses considered were: 1) disseminated intravascular coagulopathy; 2) heparin-induced thrombocytopenia (HIT); and 3) vancomycin-induced thrombocytopenia (VIT). Disseminated intravascular coagulopathy was ruled out because his prothombin time, fibrinogen level, D-dimer level and peripheral smear were all normal. Blood cultures also showed no growth. HIT was excluded by the absence of heparin-induced anti-platelet antibodies. A vancomycin-induced anti-platelet antibody test revealed IgG-positive antibodies detected in the presence of vancomycin. IgM antibodies were negative.

## Discussion

**Figure 1:**
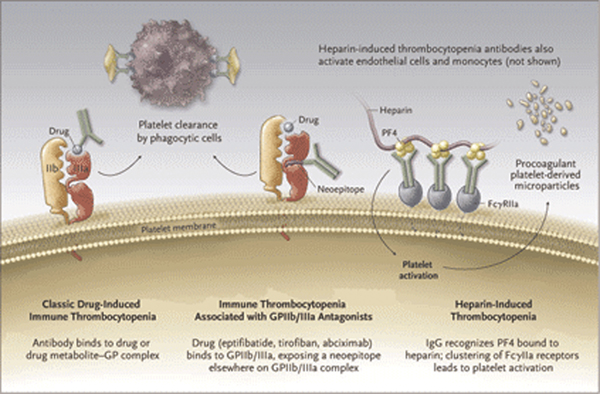
**Mechanism of drug induced thrombocytopenia**.

In general, drugs can cause thrombocytopenia by direct toxic effect, hapten formation and innocent bystander immune response [1,15] (Figure 1).

### Direct toxic effect

Examples of this response include bone marrow destruction by myelotoxicity of chemotherapeutic agents [1].

### Innocent bystander

In this category, a drug combines with a specific antibody and is adsorbed into the platelet membrane [1]. An example of this type is heparin-induced thrombocytopenia [1]. In contrast with hapten mediated thrombocytopenia (i.e., VIT), the degree of thrombocytopenia in HIT is moderate, as the nadir-platelet count is 60,000/mm^3^[15] among 85% to 90% of patients studied,

### Hapten formation

In this type of response, (anti-platelet) antibody binds to drug (vancomycin) or to the drug metabolite-glycoprotein (glycoproteinIIb-IIIa) complex, [1,15] platelets bear thousands of copies of glycoprotein IIb/IIIa [15]. These antibody-coated platelets are cleared from circulation by macrophages of the mononuclear-phagocytic system, which recognize the "Fc tail" of drug-dependent antibodies [15]. In about 85% to 90% of patients studied, the nadir-platelet count is less than 20,000/mm^3^[15]. This increases the risk of bleeding and hemorrhage [15]. Examples include immune thrombocytopenia, following the use of vancomycin or quinine [15].

### Features of VIT

Prominent features of VIT have been described in recent studies. [5,15] Patients are usually exposed to vancomycin for at least six days. Platelet counts drop by a mean of 93% from pre-treatment value. Nadir counts averaging 13,600/mm^3^ are reached about eight days after vancomycin treatment (the range is one to 27 days). Platelet counts return to pre-treatment values after discontinuation of the drug. One-third of patients have extensive ecchymoses and hemorrhage (wet purpura). Detection of vancomycin-induced IgM antibodies in one of 451 normal subjects raises the possibility that in rare cases, naturally occurring antibodies may cause acute thrombocytopenia after a single dose of vancomycin. However, vancomycin-induced IgG antibodies were not detected in any of 451 normal subjects studied.

Other features of VIT include the failure of platelet transfusion to elevate platelet levels in most patients [5] (77% to 79% in that study). Vancomycin-induced antibodies can persist for months following exposure to vancomycin. Thrombocytopenia in patients with renal failure can persist long after vancomycin medication is discontinued, presumably because of delayed clearance.

## Conclusions

The diagnosis of drug-induced thrombocytopenia is often overlooked. However, it is a potentially life-threatening condition, and testing with drug-dependent antibodies, such as vancomycin-induced platelet-reactive antibodies, can be helpful in identifying the cause of thrombocytopenia in patients receiving vancomycin [5].

## Abbreviations

HIT: heparin-induced thrombocytopenia; PICC: peripherally inserted central catheter; VIT: vancomycin-induced thrombocytopenia.

## Competing interests

The authors declare that they have no competing interests.

## Consent

Written informed consent was obtained from the patient for publication of this case report and any accompanying images. A copy of the written consent is available for review by the Editor-in-Chief of this journal.

## Authors' contributions

RAS was a major contributor in analyzing patient data and in writing the manuscript. AM contributed in reviewing the literature and in acquiring data. NK revised the manuscript critically for important intellectual content. All authors read and approved the final manuscript.
